# Effect of wildfire on the prevalence of opioid misuse through anxiety among young adults in the United States: A modeling study

**DOI:** 10.21203/rs.3.rs-3940689/v1

**Published:** 2024-02-20

**Authors:** Sigal Maya, Ali Mirzazadeh, James G. Kahn

**Affiliations:** University of California San Francisco; University of California San Francisco; University of California San Francisco

**Keywords:** wildfire, substance use, opioid use, anxiety

## Abstract

**Background:**

Exposure to climate change events like wildfires can lead to health and mental health problems. While conceptual frameworks have been hypothesized describing the potential relationship between disaster exposure and substance use, the association remains under-researched and unquantified.

**Methods:**

We constructed a quantitative portrayal of one proposed conceptual framework that focuses on the intermediary role of anxiety. We used the Monte Carlo simulation to estimate the impact of wildfire exposure on opioid misuse outcomes through increased anxiety. We searched for and extracted prior empirical evidence on the associations between wildfire anxiety and anxiety-opioid misuse. A base case scenario (S1) was devised in which the impact of wildfire on opioid misuse was limited to increasing anxiety incidence. Two exploratory scenarios investigated the additive roles of altered anxiety phenotype (S2) and increased severity of pre-existing anxiety (S3) due to wildfire exposure.

**Results:**

Models show that the prevalence of opioid misuse post-wildfire may rise to 6.0%–7.2%. In S1 (base case), the opioid misuse prevalence ratio was 1.12 (95% uncertainty interval [UI]: 1.00 – 1.27). The two exploratory scenarios, with less stringent assumptions, yielded prevalence ratios of 1.23 (95% UI: 1.00 – 1.51) and 1.34 (95% UI: 1.11 – 1.63).

**Conclusions:**

Our modeling study suggests that exposure to wildfires may elevate opioid misuse through increasing anxiety incidence and severity. This may lead to substantial health burdens that may persist long after the initial wildfire event, which may offset recent gains in opioid misuse prevention.

## INTRODUCTION

Over the past few years, a growing research base has demonstrated a tremendous impact on mental health related to climate change and extreme weather events. [1–8] The chronic exposure to climate change, such as witnessing changing landscapes, increasing temperatures, and altered weather patterns have been shown to lead to emotional distress broadly, described as eco-anxiety, or solastalgia. [7, 8] On the other hand, acute experiences like wildfires, storms, and heatwaves also lead to poor emotional well-being. Depression, anxiety, post-traumatic stress, and a slew of other mental health outcomes have been found to increase in communities affected by such acute experiences of climate change, in a wide array of settings across the globe. [7–10]

Some studies looked at substance use as another health outcome affected by exposure to extreme weather events such as storms, hurricanes, and wildfires. [9, 11–13] Though quantitative evidence is limited, narrative reviews as well as anecdotal evidence have led to the development of several conceptual frameworks describing the relationship between climate change exposures and substance use. [14, 15] Potential mechanisms include changing drug use patterns and access to substances due to altered physical environment, and via poor mental health (e.g., depression, stress, anxiety) in the aftermath of a disaster. [11, 16, 17] Such events may disrupt communities and reduce access to drug treatment services which may further leave individuals vulnerable to poor substance use outcomes. [18, 19]

Taken together, the available evidence might be hinting to a brewing syndemic of climate change and substance use. One meta-analysis found that for each 1 C increase in temperature, the risk for substance-related mortality increases 4.6%. [20] In 2021, 60% of heat-associated deaths in Maricopa County, Phoenix, also involved substance use. [21] In Canada, those exposed to the Fort McMurray fire in 2016 had almost three times the prevalence of substance use disorders one year later, compared to the national average. [22] Increases in substance abuse were seen similarly in Australian young adults exposed to bushfires. [23] This is concerning for the U.S., where the annual death rate for opioid use disorder is 14.9 per 100,000, and opioid use disorder morbidity and mortality cost over $1 trillion each year. [24, 25]

In this modeling analysis, we aimed to quantify the impact of wildfires on opioid misuse in the U.S., through the possible mediating path of anxiety. Shedding light on the magnitude of this association can inform future research on this topic and can help guide public health resources to where they are needed.

## METHODS

**Figure 1:**
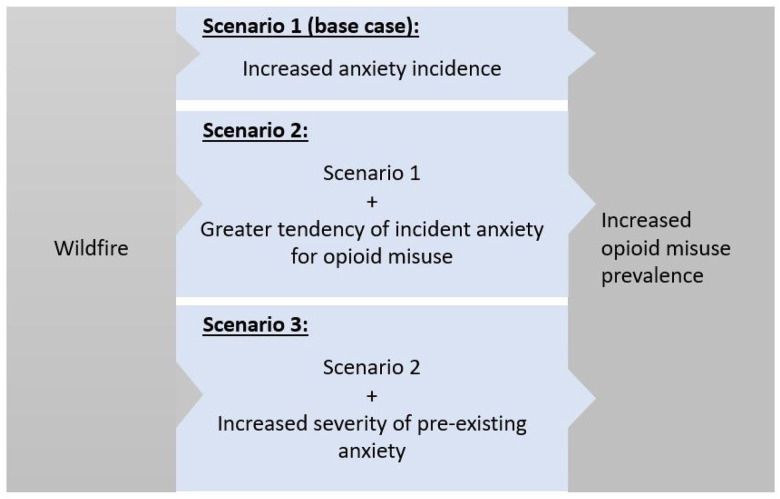
Conceptual framework of the relationship between wildfire experience, anxiety, and opioid misuse.

We quantitatively portrayed the hypothesized pathway from wildfire exposure to increased opioid misuse via increased anxiety incidence and severity. [15] We used a Monte Carlo simulation to probabilistically estimate opioid misuse outcomes as a result of experiencing a wildfire event. We focused on U.S. young adults (ages 18–25) among whom the prevalence of opioid misuse is 40% greater than the national average. [26] The model was parameterized using empirical evidence on the impact of wildfire exposure on anxiety and anxiety on opioid misuse, obtained via literature review. The model was calibrated to the overall prevalence of opioid misuse in young adults in the U.S. [26] In Scenario 1 (S1, base case), we limited the impact on opioid misuse to the rising incidence of anxiety as an outcome of wildfires. We then devised two additional exploratory scenarios to estimate modified relationships within the conceptual framework (Fig. 1). Scenario 2 (S2) hypothesized that exposure to wildfires would not only increase the prevalence of anxiety but also cause an altered anxiety phenotype with a greater tendency for opioid misuse. Scenario 3 (S3) additionally included the worsening of pre-existing anxiety following experiencing a wildfire.

Two variables of interest, the odds ratio for opioid misuse given fire-related anxiety and the prevalence of anxiety given wildfire exposure, were defined as log-normal and beta distributions, respectively. Distribution parameters were determined by visually approximating the histograms of each distribution to the available range of empirical values (Table 1). The simulation was run for 50,000 iterations to produce distributions of two outcome variables of interest: the overall prevalence of opioid misuse after a wildfire event, and the respective prevalence ratio for opioid misuse among those exposed to a wildfire. The model was implemented in R version 4.1.3. [27]

## RESULTS

**Figure 2:**
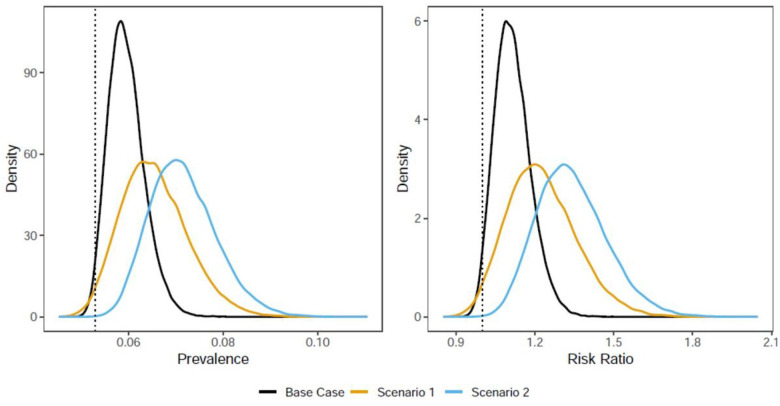
Probability density functions for the prevalence (left) and risk ratio (right) for opioid misuse among those exposed to a wildfire. Scenario 1 limits the impact of wildfire on opioid misuse to increasing anxiety incidence. Scenario 2 additionally incorporates a greater tendency for opioid misuse in incident anxiety. Scenario 3 further incorporates increased severity of pre-existing anxiety.

The model suggests increasing opioid use prevalence following exposure to wildfire (Table 2, Fig. 2). Exposure to a wildfire event led to a persistent increase in the prevalence of anxiety (prevalence ratio 1.98; not shown). In S1, this increase led to an opioid misuse prevalence of 6.0%, reflecting a prevalence ratio (PR) of 1.12 (95% uncertainty interval [UI]: 1.00–1.27).

In S2, we assumed exposure to wildfire was not only a predictor of anxiety, but also that wildfire-associated anxiety was more likely to lead to opioid misuse. Given a hypothetical risk ratio of 5 for opioid use given wildfire-induced anxiety, the prevalence of opioid use increased further to 6.5% (PR 1.23, 95% UI: 1.00–1.51). S3 further considered the impact of wildfire exposure on pre-existing anxiety, wherein wildfires increased the severity of anxiety and made it more likely for those with pre-existing anxiety to start using opioids. In this scenario, we estimated opioid use prevalence as 7.2% (PR 1.34, 95% UI: 1.11–1.63).

## DISCUSSION

We estimated the magnitude of the effect of wildfire exposure on opioid misuse among young adults in the U.S. Our model showed that exposure to a single wildfire event may substantially elevate the prevalence of opioid misuse, ranging from a 12–34% increase. The magnitude of this association depends on the extent to which wildfire exposure alters anxiety incidence and severity.

Opioids present a significant problem in the U.S. with large health and financial burden, all of which can be exacerbated by wildfires. As expected, our findings indicate greater prevalence of anxiety post-wildfire, and in turn greater prevalence of opioid misuse, even in the most limiting assumptions of the base case scenario. This has long-term implications for the health of the U.S. population. While the immediate dangers of experiencing a wildfire are relatively transient, mental health problems like substance use triggered by such an experience may persist for a long time after the wildfire subsides, leading to heightened morbidity and mortality, and associated costs, for many years to follow. [22, 29–31] Consequently, increasing frequency of wildfires and other climate disasters may stunt the gains achieved over the last decade in opioid misuse prevention. [26] This highlights the potential of climate change adaptation and mitigation as primary opioid misuse prevention mechanisms.

It is noteworthy that the prevalence ratios for the base case (S1) and S2 include 1.00, suggesting there may be no change in opioid misuse prevalence after wildfire exposure compared to baseline under these scenarios. While counterintuitive, a disaster or extreme weather event may lead to a reduction in substance use, offsetting increases in substance use caused by the same. For instance, those in disaster-affected areas may be evacuated to other settings where they are unfamiliar with local drug access mechanisms, and the lack of privacy in public disaster shelters may discourage use. Greater community support as well as personal resilience factors may also attenuate the negative impacts of a disaster on mental health, including substance use. [32] Additionally, individuals may reduce substance use as a way to adapt to the altered social environment after the event. [33]

The dearth of empirical evidence on this relationship led us to opt for a probabilistic model which incorporates uncertainty around key parameters that drive results: prevalence of anxiety following wildfire, and the consequent increase in opioid misuse. By defining probability distributions aligned with available evidence for these two parameters, no matter how limited, we were able to produce a range of plausible opioid misuse outcomes which overall indicate a harmful effect from wildfire exposure.

Our findings should be considered in light of several limitations. First, a key model data point on the relationship between wildfire exposure and anxiety was obtained from a set of studies conducted in Canada, where the government response and community support following the wildfire might have been different than in the U.S. It is generally understood that greater support after a disaster is associated with lessened harmful impacts of that disaster. [34] Second, we relied on a simplified pathway from wildfire to opioid misuse, singling out the role of anxiety alone. In reality, it is likely that more complex mechanisms will be involved, with other intermediary outcomes that may change the prevalence of opioid misuse in either direction, though the overall impact is likely to be negative. Similarly, our portrayal of a single wildfire event may have led us to underestimate health harms. As large-scale natural disasters become more frequent, individuals are at risk for cumulative mental health impacts associated with experiencing subsequent extreme weather events. [35]

Despite the limitations, our evaluation suggests that experiencing a wildfire can increase opioid misuse prevalence as much as by a third through increased anxiety incidence and severity, possibly offsetting recent improvements. This warrants further research on opioid misuse outcomes related to wildfires to better understand the impact and whether a downward trajectory in opioid misuse can be maintained given climate change stressors. Future work should focus on identifying and quantifying other possible causal pathways and determine sub-groups who may experience heightened vulnerability to negative mental health impacts, so that timely and appropriate interventions may be designed and implemented.

## Figures and Tables

**Table 1:** Model parameters

Parameter	Mean Value	Source	Notes
***Static parameters***			
Prevalence of opioid misuse	5.3%	SAMHSA 2020 [26]	Past-year prevalence of opioid misuse among U.S. youth aged 18–25.
Prevalence of anxiety	6.6%	Global Burden of Disease Study 2019 [28]	Past-year prevalence of anxiety in the U.S. among those aged 20–24.
Risk ratio for opioid use among those with anxiety in Scenario 1	5	Hypothetical	Assumes higher risk ratio to reflect pre-disposition to opioid misuse.
Risk ratio for opioid use among those with anxiety in Scenario 2	5	Hypothetical	Assumes higher risk ratio to reflect increased opioid misuse risk among pre-existing anxiety.
**Probabilistic parameters**			
Odds ratio for opioid misuse given fire-related anxiety	Log-normal distribution with mean 3.04 and standard deviation 1.07	Agyapong 2018, [17] Moosavi 2019, [11] Ritchie 2021, [16] authors’ calculation	Mean value is the median of 3 studies conducted by the same research group in Fort McMurray, Canada, following the 2016 wildfires. All 3 studies measured anxiety using GAD-7 6–18 months post-fire in different sub-populations.
Anxiety prevalence among those exposed to wildfire	Beta distribution with alpha 10.5 and beta 70	Belleville 2021, [22] authors’ calculation	Likely anxiety (measured via GAD-7) among Fort McMurray residents one year after the wildfire. Nation-wide prevalence was 3%.

**Table 2:** Mean results of the Monte Carlo simulation (50,000 iterations).

	Opioid misuse prevalence	Prevalence ratio for opioid misuse post-wildfire
	(95% UI)	(95% UI)
**Scenario 1 (base case; more prevalent anxiety)**	6.0% (5.3% - 6.8%)	1.12 (1.00–1.27)
**Scenario 2 (opioid pre-disposed anxiety)**	6.5% (5.3% - 8.1%)	1.23 (1.00–1.51)
**Scenario 3 (worsening pre-existing anxiety)**	7.2% (6.0% - 8.7%)	1.34 (1.11–1.63)

UI: Uncertainty interval

## Data Availability

All data generated and/or analyzed during this study are included in this published article and its references.
